# Acute chest pain: Acute coronary syndrome versus lead perforation: A case report

**DOI:** 10.1186/1755-7682-3-13

**Published:** 2010-07-06

**Authors:** Prashanth Peddi, Deepthi Vodnala, Jagadeesh K Kalavakunta, Ranjan K Thakur

**Affiliations:** 1 Michigan State University/Sparrow Health System, Lansing, Michigan, 48912, USA; 2 Michigan State University/Kalamazoo Center for Medical Studies, Kalamazoo, Michigan, 49008, USA

## Abstract

**Background:**

Diagnosing pacemaker lead perforation in the setting of chest pain and EKG changes is difficult and usually not considered unless we have awareness and high index of suspicion. This kind of clinical scenario represents one of the diagnostic challenges.

**Case presentation:**

A 77 year-old Caucasian female came to emergency room with left sided non-exertional chest pain radiating to her back for the past two days. A week prior to this presentation, she had a stent supported angioplasty for in-stent re-stenosis and subsequently dual chamber pacemaker implantation for sick sinus syndrome. On physical exam she is very obese, had normal vital  signs, peripheral pulses and cardio-respiratory exam. Electrocardiogram revealed new T- wave inversions in inferior and anterior leads. Initial chest X-ray, 2D-Echocardiogram and cardiac enzymes were normal. Acute coronary syndrome was considered as an initial probable diagnosis. She was anticoagulated with heparin and eptifibatide. Patient continued to have chest pain with negative cardiac biomarkers. She developed hypotension, oliguria, elevated white count, pyuria and renal failure. Because of a normal 2D-echocardiogram, cardiac etiology  for shock was not suspected.   After initial fluid challenge, empiric treatment for septic shock was initiated with antibiotics and vasopressors. Work up for pulmonary embolism and intra-abdominal hemorrhage was negative. Because of persistent chest pain, shock with cold &  clammy extremities and elevated central venous pressure cardiogenic  shock was considered and a repeat 2D-echocardiogram was done on third  day of hospitalization which revealed pericardial effusion.   Non-contrast CT-scan chest done to look for lead position confirmed that she had hemorrhagic pericardial effusion along with lead perforation. Patient underwent pericardial window placement along with over-sewing of atrial wall to seal the leakage point. The patient improved and was then discharged from the hospital.

**Conclusion:**

Lead perforation presenting with chest pain and EKG changes is often not appreciated resulting in significant delay in diagnosis and inappropriate treatment.

## Background

Diagnosing pacemaker lead perforation in the setting of acute chest pain and EKG changes is very difficult and usually not considered unless we have awareness and high index of suspicion. This kind of clinical scenario represents one of the diagnostic challenges in medicine.

## Case presentation

A 77 year old Caucasian female presented to emergency room on 09/06/2009 with left sided chest pain of three days duration. Her pain was sharp, 8/10 in intensity, nonexertional, radiated to interscapular region, and initially relieved by over the counter medications. She denied any fever, cough, nausea, vomiting, diaphoresis, shortness of breath and, palpitation. Other review of systems was unremarkable.

Past medical history included hypertension, dyslipidemia, obstructive sleep apnea, atrial fibrillation and coronary artery disease. She had bare metal stent placement in left anterior descending artery in 06/09, angioplasty for in stent restenosis in 08/09 and a pacemaker implantation for sick sinus syndrome on 09/01/2009. Her medications included lisinopril, atenolol, simvastatin, aspirin, clopidogrel, warfarin, and dofetilide.

On physical examination she was morbidly obese, was in moderate distress. Her vital signs included temperature of 98.7°F, pulse rate 63/minute, regular in rate and rhythm, blood pressure (BP) 141/62 mmHg, respiratory rate (RR) 18/min SpO_2 _97%, jugular venous pressure (JVP) was not elevated and there was no pulsus paradoxsus. Auscultation of heart and lungs was unremarkable. Examination of other systems was normal.

**Figure 1:**
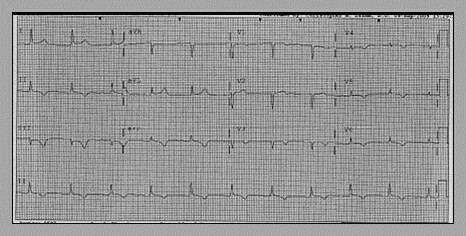
EKG-T wave inversions in II, III, AVF and QS complexes, T wave inversions in V1-V6.

Admission electrocardiogram (EKG) revealed new T wave inversions in the lead II, III, AVF and QS complexes and T wave Inversions in V1-V6 as seen in the previous EKG (Fig. 1). Admission chest X-ray showed normal cardiac silhouette with presence of pacemaker leads, normal lung fields and mediastinum.

Her initial set of cardiac enzymes, complete blood count (CBC), basic metabolic panel (BMP) and urine analysis were normal. She was admitted with diagnosis of unstable angina. Treatment with heparin, eptifibatide and nitroglycerine was begun. Her repeat EKG on 09/07/09 showed ST depressions in the inferior leads, repeat cardiac enzymes were normal, however her chest pain continued to persist. 2D Echocardiogram (ECHO) on 09/07/09 revealed ejection fraction (EF) of 65%, without any pericardial effusion. Chest pain continued to persist, became hypotensive and oliguric, blood pressure medications were with held. Repeat CBC and BMP revealed elevated WBC 15,800/μl and hemoglobin of 9.4 gm/dl. Serum creatinine changed from 1.13 to 3.01 and blood urea nitrogen increased from 29 to 53. Urine analysis revealed 15-20 white blood cells per high power field (HPF), positive leukocyte esterase and nitrite, 5-10 red blood cells per HPF.

**Figure 2:**
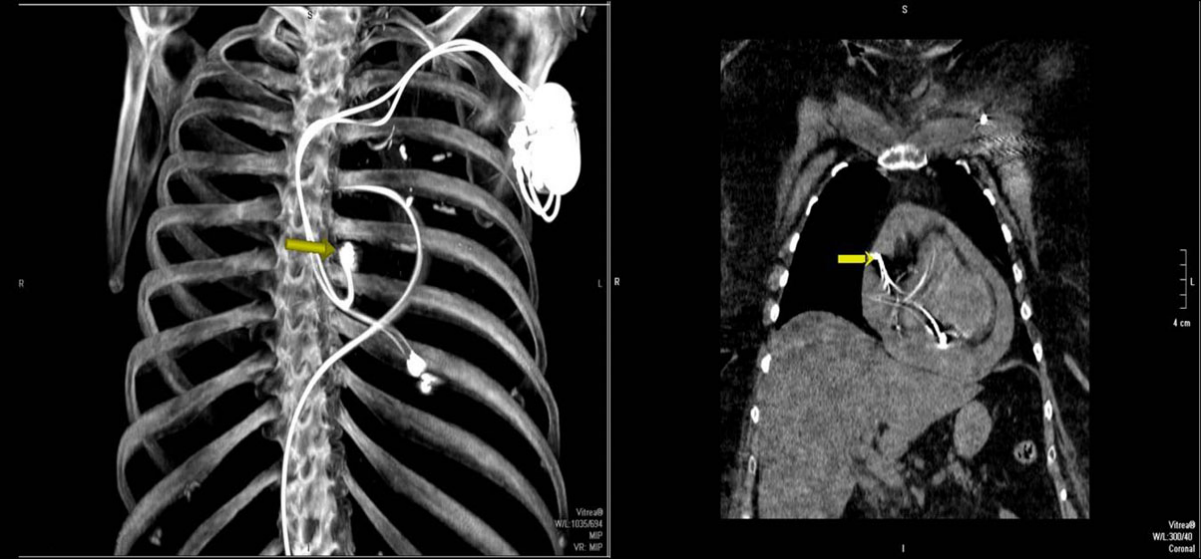
**Multiplanar reformatted images from unenhanced CT of the chest demonstrate dense pericardial fluid consistent with hemopericardium**. The atrial lead (yellow arrow) is noted to extend into the pericardial fluid (upper right image) with imaging limited due to motion artifact and streak artifact. Note: The right ventricular lead can also be seen on this coronal image which appears to be within the wall of the right ventricle. Maximum intensity projection image (lower left image) demonstrates the presence of a dual-lead pacemaker as well as a femoral-route pulmonary artery catheter.

**Figure 3:**
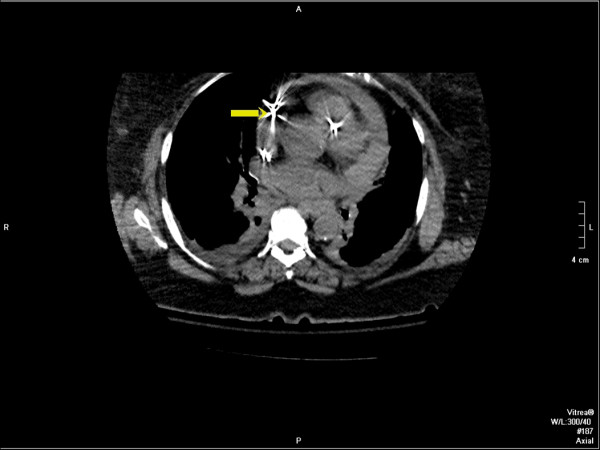
**CT scan coronal reformatted image from an unenhanced CT of the chest demonstrates dense pericardial fluid consistent with hemopericardium**. Note the pericardial fluid is isodense to the blood in the left ventricle.

**Figure 4:**
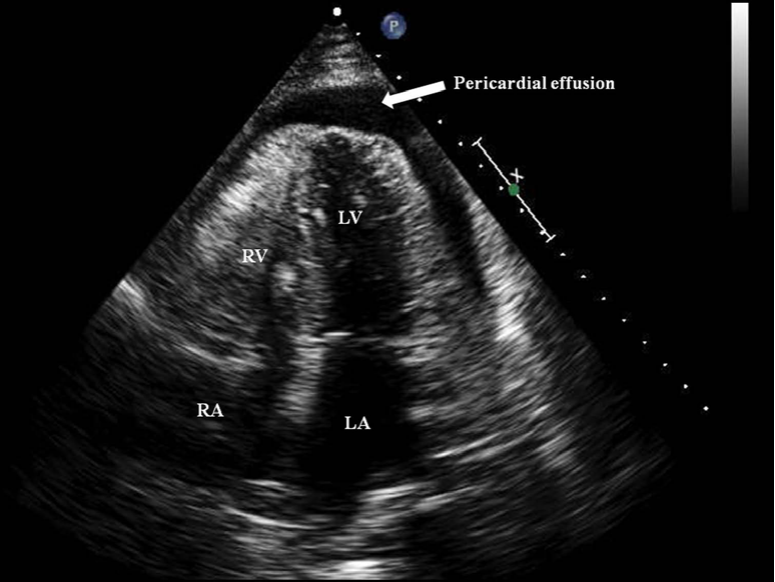
Transthoracic echocardiogram Apical 4 chamber views showing the pericardial effusion.

In view of leucocytosis, increased renal parameters and pyuria septic shock secondary to urinary tract infection was considered and transferred to Intensive care unit (ICU). After fluid resuscitation, broad spectrum antibiotics were intiated. On 09/09/09 in ICU the patient was in distress and continued to have chest pain. Vital signs were significant for pulse rate 90/min, regular, BP 70 mmHg, RR of 22/min, SpO2 95%. Auscultation of heart and lungs was unremarkable. Central venous pressure was 15 cm of water. Intravenous vasopressor therapy was intiated. As her chest pain continued to persist, a non-contrast computerized tomography (CT) of the chest was done which revealed hemorrhagic pericardial effusion (Figs. 2 and 3) with lead perforation of the right atrium which was subsequently confirmed by 2D ECHO (Fig. 4)

She underwent pericardial window for drainage and evaluation of lead perforation. During surgery the screw in right atrium had gone through the right atrial wall and caused the perforation. Surgeons over sew the atrial wall and drained 200 cc of pericardial fluid. Her post operative recovery was uneventful and discharged home on the fifth day.

## Discussion

Pacemaker implantation is a fairly benign procedure. Myocardial perforation is a rare complication. Contrary to the ventricle where pacing leads remain passively implanted, the use of active screw-in atrial pacing leads has rapidly developed during the last few years [1].

This type of fixation in a thin and fragile cardiac wall carries a risk of perforation and thereby of pericardial complications. Acute perforation of the myocardium during the placement of pacemaker leads happens in 1%-7% of patients [2]. Late perforation of the pacemaker leads is a further rare complication and it is associated with varied time frame from days to months.

Atrial perforation symptoms depend on the displaced electrode position, sensing threshold, failure of pacing, development of pericardial effusion and involvement of the phrenic nerve. Development of pericardial tamponade with atrial screw leads may result from direct perforation, irritation or slow oozing of the pericardium [3]. Ventricular perforations spontaneously seal with the retraction of the pacing wire, but the atrial perforations almost always require surgical intervention as in this case.

Imaging studies including chest x-rays, 2D/3D ECHO and CT scan have been utilized to diagnose the atrial perforation. The superiority of the either CT scan or ECHO has to be further studied [4]. In this case the suspicious chest x-ray after the placement of the central line lead to the further investigations. Persisting chest pain without elevation of troponins and hemodynamic instability with elevated JVD raised the suspicion pericardial tamponade. CT scan of the chest confirmed the hemorrhagic pericardial effusion along with the exact position of the right atrial lead. 2D ECHO with multiple projections further confirmed the effusion. During the surgery the lead was noted to perforate the right atrium and it was retrieved and the perforated atrial wall was repaired.

## Conclusions

1. This case report enlightens the importance of consideration of perforation in patients who presented with acute chest pain and a past history of pacemaker placement before anticoagulation is begun to avoid catastrophic complications.

2. A normal initial 2D ECHO does not rule out cardiogenic shock; however if the index of suspicion is high and if the clinical features such as elevated JVP and signs of shock are present repeat 2D ECHO should be considered to make accurate diagnosis.

## Competing interests

The authors declare that they have no competing interests.

## Authors' contributions

All the authors are involved in the conception and design, acquisition of data, or analysis and interpretation of data; drafting the article or revising it critically for important intellectual content; and final approval of the version to be published.

## Consent

Written informed consent was obtained from the patient for publication of this case report and accompanying images. A copy of the written consent is available for review by the Editor-in-Chief of this journal.
